# Evolution of subtype C HIV-1 Env in a slowly progressing Zambian infant

**DOI:** 10.1186/1742-4690-2-67

**Published:** 2005-11-07

**Authors:** Hong Zhang, Federico Hoffmann, Jun He, Xiang He, Chipepo Kankasa, Ruth Ruprecht, John T West, Guillermo Orti, Charles Wood

**Affiliations:** 1 Nebraska Center for Virology, University of Nebraska, Lincoln, NE, USA; 2 The School of Biological Sciences, University of Nebraska, Lincoln, NE, USA; 3 Department of Pediatrics, University Teaching Hospital, Lusaka, Zambia; 4 Dana-Farber Cancer Institute, Harvard Medical School, Boston, MA, USA

## Abstract

**Background:**

Given the high prevalence of mother to child infection, the development of a better understanding of African subtype C HIV-1 transmission and natural evolution is of significant importance. In this study, we genotypically and phenotypically characterized subtype C viruses isolated over a 67-month follow-up period from an *in utero*-infected Zambian infant. Changes in genotype and phenotype were correlated to alterations of the host humoral immune response.

**Results:**

A comparison of baseline maternal and infant samples indicated that the infant sequences are monophyletic and contain a fraction of the diversity observed in the mother. This finding suggests that selective transmission occurred from mother to child. Peaks in infant HIV-1 Env genetic diversity and divergence were noted at 48 months, but were not correlated with changes in co-receptor usage or syncytia phenotype. Phylogenetic analyses revealed an accumulation of mutations over time, as well as the reappearance of ancestral lineages. In the infant C2-V4 region of Env, neither the median number of putative N-glycosylation sites or median sequence length showed consistent increases over time. The infant possessed neutralizing antibodies at birth, but these decreased in effectiveness or quantity with time. *De novo *humoral responses were detected in the child after 12 months, and corresponded with an increase in Env diversity.

**Conclusion:**

Our study demonstrates a correlation between HIV-1 Env evolution and the humoral immune response. There was an increase in genetic diversification in the infant viral sequences after 12 months, which coincided with increases in neutralizing antibody titers. In addition, episodes of viral growth and successive immune reactions in the first 5–6 years were observed in this slow progressor infant with delayed onset of AIDS. Whether this pattern is typical of slow progressing subtype C HIV-1 infected infant needs to be further substantiated.

## Background

Subtype C human immunodeficiency virus type 1 (HIV-1) accounts for over 56% of HIV-1 infections [1-3]. Globally, HIV-1 infection is one of the leading causes of childhood morbidity and mortality. HIV-1 infected children account for 20% of all HIV-1 related deaths; 7% of individuals living with HIV-1 infection, and 16% of new HIV-1 infections annually [4]. In sub-Saharan Africa, HIV-1 subtype C is responsible for approximately 50% of infections and a significant number of infections are in infants and children. Transmission of HIV-1 from infected mothers to their infants is the primary mode of HIV-1 infection in children and can occur *in utero*, intrapartum, or postnatally through breast milk. The use of antiretroviral regimens has successfully reduced the rate of HIV-1 infection in infants in the developed world to approximately 1%; nevertheless, such regimens have only recently become available in many of the developing nations where HIV-1 mother to child transmission (MTCT) is most significant [5].

HIV-1 MTCT is complex, and its determinants are not completely understood. Several factors, including high maternal viral load, maternal *env *gene homogeneity, and rapid viral replication kinetics, have been correlated with perinatal HIV-1 transmission [6-8]. In addition, advanced maternal disease status, lack of drug therapy, and lack of breast-feeding alternatives contribute to increased MTCT [9]. Moreover, several studies have demonstrated the transmission of minor [9-12], major [9,11], and multiple [9,13,14] HIV-1 genotypes from mother to infant. Our understanding of perinatal transmission and disease progression in infants is mainly derived from studies of subtype B infected individuals. The applicability of such findings to other subtypes remains to be substantiated.

The natural history of subtype C HIV-1 infection has not been extensively studied in children. It is known that infant disease survival times are considerably shorter than those of HIV-infected adults, and that without treatment, most HIV-1 infected African children die before their third birthday [15]. Given the expanding distribution of subtype C infections, a complete understanding of virus transmission and natural evolution is increasingly important.

HIV-1 transmission is, in part, a function of the receptor binding by the envelope glycoprotein (Env) that mediates virus-cell fusion. Alteration of Env has been linked to expanded host range, alternative co-receptor usage and *in vitro *syncytium induction and associated with viral pathogenesis and disease progression [16-25]. Accumulating evidence suggests that subtype C Env displays biological properties, such as near-exclusive CCR-5 utilization, that distinguish it from other subtypes. In addition, the subtype C Env glycoprotein, third variable region (V3) is more conserved than the previously defined "constant" regions [26,27]. Whether differences in cellular tropism, transmission and pathogenetic outcome observed between subtype C and other subtypes correlate with the Env glycoprotein biological or genetic properties need to be examined. In addition, whether there exist differences in Env evolution in infected children based on viral subtype, remains to be determined. Recently it has been suggested that particular changes in *env *in Zambian adults correlated with heterosexual transmission. Viruses with shorter Env length, and fewer putative N-linked glycosylation sites (PNGS) were suggested to be more susceptible to neutralizing antibodies, yet more efficient at transmission [28]. Similar correlates have not been reported for transmission to children.

In the present study, we investigated the longitudinal variation of the viruses in a subtype C HIV-1 infected Zambian mother/infant pair (MIP 1157). This pair was antiretroviral therapy naïve over a six-year follow-up period. The extended follow-up enabled us to examine the interplay between humoral immune selection and virus evolution. We describe changes in the infant Env C2-V4 region over the follow-up period, and correlate these changes with alterations in viral phenotype and host humoral immune response. Our findings indicate that genetic diversification in the infant Env gene increased after 12 months, and is correlated with increases in neutralizing antibody titers.

## Results

### HIV-1 infected mother-infant pair

We characterized HIV-1 transmission and longitudinal evolution of the HIV-1 envelope glycoprotein in a Zambian mother and infant pair (MIP 1157) for more than 6 years. The mother and child are anti-retroviral naïve and remain clinically asymptomatic. Infant 1157 was infected *in utero *since HIV-1 sequences were detected by DNA PCR of infant blood samples collected at birth. The baby was delivered naturally, healthy and with normal birth weight, and was breast-fed until 20 months of age. The child remains clinically asymptomatic throughout the follow-up study period and his CD4 counts was 658 cells/μl at 6 years old. The child has been evaluated at the study clinic where blood specimens were collected every 6 months for the first 24 months and at 12-month intervals thereafter. The prolonged survival of this infected child is unusual since most untreated HIV-1 infected African children do not survive beyond the first three years of life. The extended follow-up of infant1157 provided us with an opportunity to investigate correlates of virus transmission in the Env glycoprotein and to track genetic variation and evolution of this gene over time.

### MIP1157 viruses use CCR5 as co-receptor and belong to subtype C

All viral isolates recovered from MIP 1157 replicated efficiently in PBMC and monocyte-derived macrophages (MDM), but failed to grow in MT-2 and C8166 T-cell lines. Viral isolates did not induce syncytia in infected PBMC and MDM. We evaluated viral co-receptor usage in cell lines that co-express CD4 with a single co-receptor. All isolates failed to infect CXCR4-expressing CEMx174-GFP cells, and similarly, none of the viruses grew in cells expressing only CCR3 (HOS-CD4-CCR3) (data not shown). In addition, 1157 viruses failed to replicate in PBMC homozygous for the Δ32 deletion variant of CCR5. In contrast, cells expressing normal CCR5 and CD4 (GHOST-CD4-CCR5) were readily infected, suggesting that 1157 HIV-1 isolates primarily use CCR5 as co-receptor (data not shown). This is in agreement with infectivity assays demonstrating that only primary PBMC and MDM support viral growth. Phylogenetic analyses clustered all 1157 *env *sequences with subtype C.

### Transmission pattern

**Figure 1:**
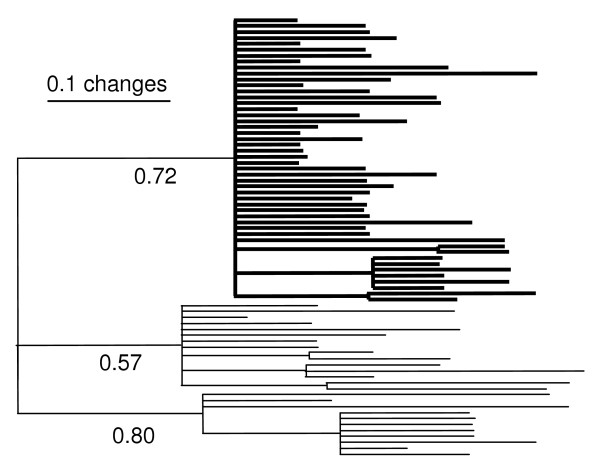
Phylogenetic relationships between mother (thin) and infant (thick) samples collected at birth. Majority rule consensus from a Bayesian analysis (BA) run for 5 × 10^6 ^generations, sampled every 1000. The last 3000 trees were used to build the consensus. Posterior probabilities are next to the relevant nodes.

Viral *env *sequences from both the mother and infant at birth were analyzed to examine the genealogical pattern of perinatal transmission. Infant birth samples were monophyletic relative to the mother in all phylogenetic analyses (Bayesian [BA], maximum likelihood [ML] and neighbor joining [NJ]). In all cases, phylogenetic trees support the concept of a restricted pattern of transmission, where a subset of the maternal quasispecies was passed into the child (Figure 1). As would be expected in a restricted transmission, genetic variation in the HIV-1 Env gene is lower in infant birth sequences than in maternal sequences from the same timepoint (Table 1). The mean number of nucleotide substitutions within the mother's *env *sequences at birth was 3.2, compared to 1.67 in the infant (Table 1), and the mean number of amino acid differences was 2 in the mother and 1 in the infant. These findings from phylogenetic and diversity analyses indicate that the infant possesses a subset of the maternal diversity at the time of birth.

**Table 1:** Viral variations in the different mother and infant populations

**Sample**	**n**	**H**	**Nuc**	**AA**	**PNGS**	**L**
Infant at birth	48	26	2 (0 – 7)	1 (0 – 5)	15 (14 – 15)	183 (183 – 183)
Infant 6 months	29	23	4 (0 – 11)	3 (0 – 9)	14 (13 – 15)	183 (175 – 183)
Infant 12 months	27	24	6 (0 – 15)	4 (0 – 11)	13 (13 – 15)	174 (174 – 183)
Infant 18 months	51	38	15 (0 – 29)	11 (0 – 23)	14 (13 – 15)	179 (175 – 183)
Infant 24 months	37	36	14 (1 – 21)	11 (0 – 18)	12 (11 – 16)	177 (174 – 183)
Infant 29 months	28	27	16 (1 – 26)	12 (0 – 19)	12.5 (11 – 15)	182 (173 – 183)
Infant 36 months	26	24	13 (0 – 27)	8 (0 – 19)	12 (10 – 14)	176 (173 – 183)
Infant 48 months	25	25	25 (2 – 37)	16 (2 – 26)	13 (12 – 14)	182 (176 – 185)
Infant 67 months	32	24	35 (0 – 57)	24 (0 – 37)	13 (12 – 15)	183 (180 – 185)
Mother at delivery	26	20	5 (1 – 10)	3 (0 – 7)	15 (14 – 15)	183 (183 – 183)
Mother 12 months	32	31	8 (1 – 23)	5 (0 – 13)	15 (13 – 15)	183 (183 – 183)
Mother 18 months	33	17	5 (0 – 13)	2 (0 – 9)	15 (13 – 15)	183 (183 – 183)
Mother 24 months	32	18	2 (0 – 7)	1 (0 – 5)	14 (13 – 14)	183 (183 – 183)

Number of samples per timepoint (n); number of unique haplotypes (H); number of nucleotide differences (nuc) as median (min-max); number of amino acid differences (AA) as median (min-max); number of putative N linked glycosylation sites (PGNS) as median (min-max), and sequence length in codons (L) as median (min – max).

### Longitudinal variation in *env *sequences

**Figure 2:**
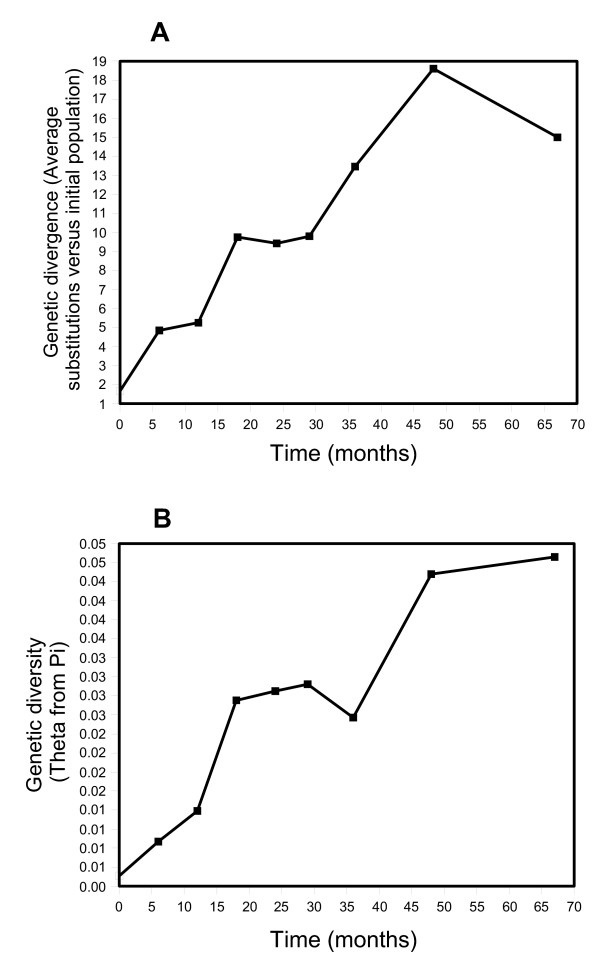
Changes in genetic divergence and diversity over time for the infant 1157. Panel A, Genetic divergence, as the average number of changes between each time point and the initial population, collected at birth. Panel B, Genetic diversity, as *θπ*, calculated from the average number of nucleotide differences within a given time point, which correlates with effective population size. The first plot describes the amount of change relative to the initial population and the second one describes the amount of variation within a time point.

Given the lack of diversity in Env from the infant birth sample, and the extended survival of the child in the absence of antiretroviral therapy, it was of significant interest to investigate evolution of the Env gene over time. Since antiretrovirals were unavailable, the primary selective pressures acting on Env from infant 1157 were maintenance of replication and immune surveillance. Population-level changes in the genetic make-up of the quasispecies within the infant were followed by measuring genetic divergence and genetic diversity over time. Genetic divergence measures the number of differences from each contemporaneous set of sequences relative to the baseline population, whereas genetic diversity is an estimate of effective population size based on the average number of pair-wise differences within each set of contemporaneous sequences. The genetic diversity and genetic divergence of the infant Env C2-V4 region increased up to 48 months, but subsequently decreased or leveled off (Figure 2).

**Figure 3:**
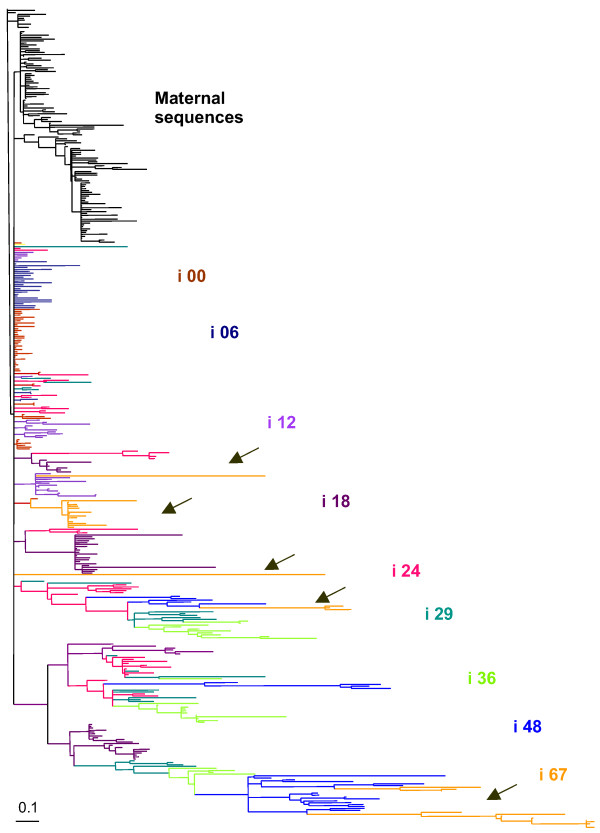
Neighbor-joining (NJ) tree describing phylogenetic relationships between mother (black) and infant (colors) samples collected from all timepoints, using a GTR model of nucleotide substitution. Labels indicate the time of collection (i. e.: i06 corresponds to sequences from the infant collected 6 months after birth).

Changes in Env genetic divergence and diversity, and in particular, the replacement of lineages over time (correlated with the stabilization of diversity and divergence), become evident when visualized in a phylogenetic tree. We constructed phylogenetic trees using NJ, ML and BA. All methods yielded similar results and only the NJ result is shown. There is an association between time of collection and sequence change (longer branches denote more changes) as early time point sequences appear on short branches, scattered at the base of the tree, while later sequences appear on long branches (Figure 3). Samples collected at 67 months are grouped into 6 different lineages, three that are closely associated with 48-month sequences, and three that are associated with sequences from earlier lineages. These would indicate that viral lineages persist in the infant and reappear at later times, e.g. some sequences collected at 67 months are closely related to sequences collected at 12, 18, and 48 months (see arrows in Figure 3). Alternatively, the virus may be selected to recreate those previous lineages as the immune pressure on particular epitopes in Env wanes.

**Figure 4:**
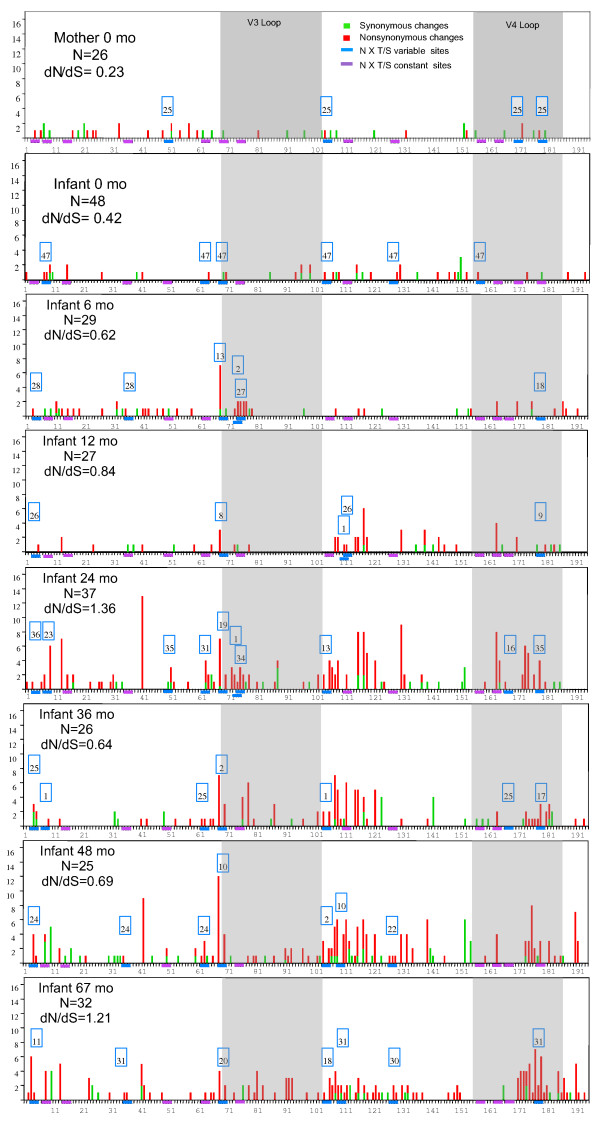
Synonymous and non-synonymous amino acids variation along the HIV- 1 Env constant region 2 (C2), variable loop 3 (V3), constant region 3 (C3), and variable loop 4 (V4). Results are presented for maternal and infant samples collected at birth, as well as for infant samples collected from 6 to 67 months. Synonymous (green) and non-synonymous (red) changes per position for each sequence set were estimated in Datamonkey. The number and position of putative N-linked glycosylation sites (PNGS) (N × T/S) was estimated in N-GlycoSite . Within each set of contemporaneous sequences, constant PNGS are indicated in purple, and variable ones with blue (with their frequency in the blue outlined box). The overall rate of non-synonymous to synonymous substitutions (dN/dS) was estimated in PAML. N: number of sequences for each timepoint.

The temporally dependent lengthening of branches seen in phylogentic trees from BA, ML and NJ analyses was similar to the idealized shape expected under continual selection. As an estimate of the relative strength of selective pressure we calculated the ratio of non-synonymous (dN) to synonymous (dS) changes (dN/dS) for each timepoint. We observed a high ratio of non-synomymous to synonymous substitutions over time as estimated by ML in PAML (Figure 4). Estimates of the overall dN/dS ratio in the infant ranged from 0.42 (i00 m) to 1.36 (i24 m). We next calculated the number of synonymous and non-synonymous substitutions per codon for each contemporaneous set of sequences to assess how selective pressure was distributed along the region of Env sequenced. Non-synonymous variation was evenly distributed in the mother and infant throughout the fragment at baseline (Figure 4). As time progressed, the number of non-synonymous changes increased in the infant Env (Figure 4), but not in the mother (data not shown). A comparison across timepoints indicates that non-synonymous variation concentrated on the first portion of the constant region 2 (C2), the first portion of the constant region 3 (C3), and the terminal portion of the variable loop 4 (V4) (Figure 4). The overall high values of dN/dS, indicated by the relative amounts of red and green in the different panels of figure 4, and tree shape (Figure 3) suggest that positive Darwinian selection is playing a strong role in shaping molecular evolution in these samples.

A recent report suggested that subtype C viruses transmitted between members of Zambian discordant couples possess envelope glycoproteins that are under-glycosylated, neutralization sensitive and contain short loop structures [28]. To explore the potential role of specific sequence characteristics in virus transmission between mother and child, we compared the sequence length polymorphism and variation in the number of PNGS for baseline maternal and infant Env C2-V4 sequences. There are 15 PNGS in this region of 1157 Env. Maternal and infant baseline sequences are all of the same length, and showed little variation in the PNGS (Table 1 and Figure 4). In the mother, there were 4 sequences, out of 26, that lost a PNGS, and the position at which this site was ablated was not conserved among any of the four. In the infant, 6 of 48 sequences lost a single PNGS, but in parallel with the mother, there was no conservation in the position of that loss. Moreover, only one variable PNGS was shared between the mother and the infant.

A similar evaluation of the C2-V4 length polymorphism and PNGS alteration was carried out on subsequent infant samples to assess the longitudinal variation in these two parameters (Table 1 and Figure 4). Length polymorphism was only observed in infant sequences where putative insertions and/or deletions occur in a subset of sequences at amino-acid positions 106–109 and 166–180. Maternal sequences remained of constant length, 183 amino acids, throughout the follow-up. All transmitted sequences in the infant were initially of the same length (also 183 amino acids). Length polymorphism in the region spanning amino acids 166–180 appeared 6 months postpartum, whereas polymorphism in the region spanning 106–109 was first observed at 12 months. The longest sequences, isolated at 48 and 67 months, were 185 amino acids in length, whereas the shortest sequences, 173 amino acids, were isolated at 29 and 36 months. All infant PNGS present at baseline remain present in a fraction of sequences from subsequent timepoints; however, only 3 sites remained fixed over the entire course of infection. The largest PNGS variation was observed at positions 7, 104, and 177, which oscillate between high and low prevalence (Figure 4, months 24, 36 and 48; position 7). In addition, there are 2 sites gained, one at position 72 at 6 months, and the other at position 109 at 12 months. Both of these polymorphisms are low in frequency and both are adjacent to another PNGS.

### Replication Kinetics

**Figure 5:**
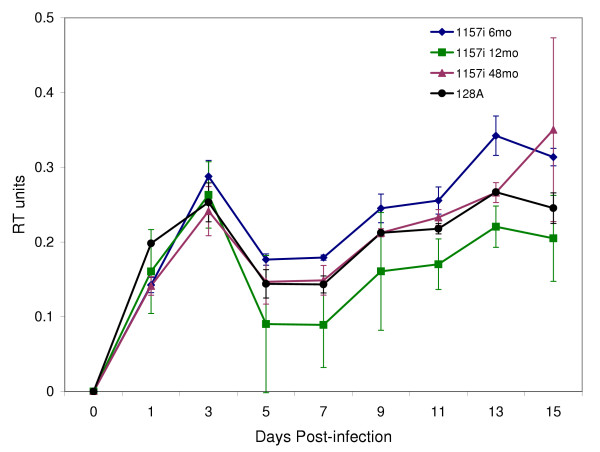
Replication kinetics of 1157 infant viral isolates obtained at 6, 12 and 48-month follow-ups were determined in PBMC culture supernatant by measuring RT units. The laboratory viral strain 128 A was used as control. Each 100 TCID_50_ viral inoculum was added to 6 × 10^6 ^PHA-stimulated PBMC from a HIV-1 seronegative blood donor. RT units were measured in culture supernatant at day 0, 1, 3, 5, 7, 9, 11, 13, 15 post-infection.

In order to determine whether there are differences in the rates of replication between early and late viral isolates, the replication kinetics of the infant isolates from 6, 12 and 48-month in primary PBMC were determined by measuring the accumulation of RT units in supernatant over time. All the viral isolates displayed similar replication kinetics with a steady increase during the first 3 days of incubation and peaked by day 3(Figure 5). The RT units dropped after 3 days and remained relatively stable for the duration of the experiment (Figure 5). In addition, the similar replication kinetics of these viral isolates was also observed in MDM (data not shown).

### Neutralization of infant HIV-1 isolates

**Figure 6:**
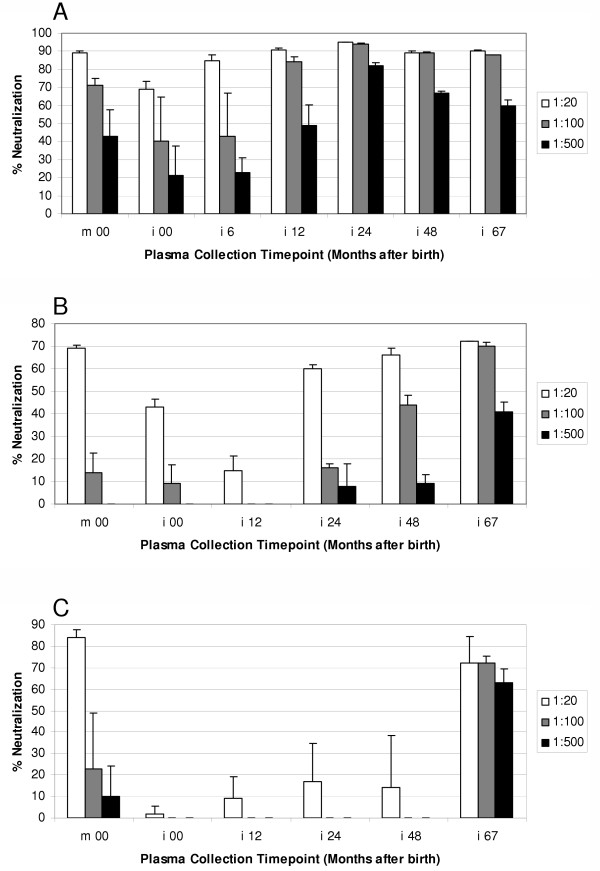
Contemporaneous and non-contemporaneous plasma neutralization activity against infant 6-month (A), 12-month (B) and 48-month (C) viral isolates, determined in TZM-BL cells. The test plasma was diluted to 1:20, 1:100 and 1:500. Virus production in the supernatants was monitored by luciferase activity 2 days post infection. Luciferase activity in the control wells containing no plasma was defined as 100 %, and the neutralization titer of the test plasma was calculated relative to this value.

To determine whether Env evolution correlated with the development of infant anti- HIV-1 humoral immunity, we analyzed neutralization of infant 6-month, 12-month and 48-month viral isolates by contemporaneous and non-contemporaneous plasma (Figure 6). The neutralization of the 6-month viral isolate by baseline infant plasma (i00) was 68% compared to 90% by baseline maternal plasma (m00) at the same dilution (1:20), indicating that only a subset of the maternal neutralizing antibody repertoire was passively transferred to the child. As expected, the ability of the contemporaneous plasma to neutralize the 6-month viral isolate (85%) was less than that achieved by 12, 24, 48 and 67-month plasmas, which achieved 91%, 95%, 89% and 90% neutralization, respectively (Figure 6A). The increase in neutralization by 6- to 67-month plasma as compared to at birth plasma suggested that *de novo *humoral immune responses against early viral genotypes persisted and became progressively stronger with time (Figure 6A). Evaluation of the contemporaneous plasma neutralization of the 12-month infant viral isolate indicated a very low level of activity (Figure 6B). Only 15% of the input virus was neutralized by the infant 12-month plasma at a 1:20 dilution; whereas, the infant plasma at birth neutralized 43%. This was 3-fold higher than the contemporaneous infant sera, but lower than the maternal plasma at delivery suggesting that most of the neutralizing antibody in the infant during the first months of life was of maternal origin. Moreover, during the first 12-month of infection, the level of neutralizing activity against the 12-month virus was observed to decrease with time indicating decay of the maternal humoral component. Thereafter, increasing titers of neutralizing antibody were detected in non-contemporaneous 24, 48, and 67-month plasma, which achieved 60, 66%, and 72% neutralization, respectively (Figure 6B). These data suggest the development of effective humoral immune responses in the infant. This increase in neutralizing humoral immunity may, in part, be responsible for observed increases in infant viral diversity during the same period. Evaluation of neutralization of the infant 48-month virus isolate revealed high titer neutralization from the maternal baseline plasma (84%), but very low level of neutralization from the infant's plasma at 24 or 48 months (Figure 6C). Nevertheless, the 67-month infant plasma neutralized 72% of the i48 m virus, suggesting a delayed but continuing infant immune response against the diversifying viral population.

## Discussion

Longitudinal evolution of HIV-1 subtype C has rarely been evaluated in infected children. The survival of infant 1157 for more than 6 years post-infection provided us with an opportunity to track genetic variation and phenotypic evolution in the viral envelope glycoprotein over that period. In addition, we were able to examine correlations between these viral properties and the humoral immune response of the child. Detection of HIV-1 sequences in PBMC collected from the child at birth indicated *in utero *infection. The pattern of genetic variation shown by phylogenetic analysis at baseline is compatible with an episode of selective transmission, as reported in previous studies [9-12,27,29]. *In utero *infection of infants has been reported to result in more rapid disease progression [30-32]; however, the extended survival of infant 1157 suggests the route of infection alone is not predictive of disease progression in subtype C infected children.

HIV-1 has replication and mutation rates that generate high numbers of progeny and significant genetic variation. The *env *gene has been calculated to diverge at a rate of about 1% per year [33]. The patterns of HIV-1 evolution in infected individuals, even for subtype B viruses, are ambiguous. Delwart et al. reported several-fold higher diversity at the early stage versus the late stage of infection [34]. In contrast, other studies have shown that viral sequences in *env *are more homogenous early in infection and diversify with disease progression and decline in CD4+ T cell counts [33,35-40]. Here we show that birth *env *sequences in the recipient child were highly homogenous, as indicated by *env *diversity, and were closely related to, but encompassed only a subset of the contemporary maternal variation. Genetic analysis at multiple timepoints showed that diversity in *env *as well as divergence from the initial infecting species increased with time up to 48 months. This increase in diversity and divergence correlated with parallel increases in non-synonymous changes. Whether such an increase is unique to this case needs to be further substantiated. Our findings contrast with those from studies of subtype B infected adults where, in patients infected with viruses that undergo co-receptor switching, the peak of diversity correlated with the development of CXCR4 utilization and the peak of divergence correlated with the maximal prevalence of CXCR4 utilizing species [33]. These phenomena are not relevant to 1157 since no alternative co-receptor usage was detected in either the mother or the child. Although X4-utilizing subtype C strains have been described [41-44], they are unusual, thus pointing to distinct evolutionary pressures on the various subtypes. Since subtype C infected individuals possess X4-expressing cells, it is likely that immunological and viral replicative selection in these individuals do not force or allow subtype C to efficiently utilize these targets or other constraints make such utilization significantly unfavorable.

Interestingly, we have observed the apparent reappearance of earlier lineages at the 67-month time point, and this is probably correlated to the decrease in viral genetic divergence at the same time point. Our observation would indicate that viral sequences, presumably emerging from latently infected cells, can reintroduce ancestral lineages and thus could lead to the decrease in divergence. It is tempting to speculate that such reintroduction might coincide with the waning of the immune response to these 'earlier' viruses in much the same way as antiretroviral therapy interruption often results in repopulation of the patient with drug-sensitive ancestral strains. Alternatively, the host environment may have altered in such a fashion that an ancestral variant becomes more viable due to higher replication fitness and decay of immune selection.

Our sequence analysis also revealed a substantial amount of variation (mutation, deletion and insertion) in *env *C3 and V4 regions in infant samples, implying that C3 or V4 domain is a likely target of immunological or replicative selective pressure during subtype C virus evolution and disease progression in children. The significance of C3 and V4 variation is currently under investigation. It is important to recognize that definitions of the constant and variable domains in Env are derived primarily from studies of subtype B viruses, and the patterns of sequence diversity in those isolates may not be reflected in other subtypes such as subtype C. 

Our neutralization assays support the concept that the humoral immune response developed in parallel with the evolving HIV-1 envelope sequences and constitutes part of the selective pressure on the gene [45,46]. The persistence of high level neutralizing antibodies against early infant viral isolates indicated that the infant immune system is capable of developing and maintaining strong responses to eliminate the initially transmitted and replicating virus (Figure 6A). It has been shown that neutralization escape mutants with reduced sensitivity to autologous sera emerge rapidly in HIV-1 infected adults [46-48], but patients subsequently developed additional neutralizing antibodies to the 'escape' viruses after a delay [49]. The initial effectiveness of the infant sera is likely due to a significant contribution by maternal antibodies to neutralization titer. Nevertheless, the child does not receive the full repertoire of maternal neutralizing antibody since a disparity was observed between the effectiveness of maternal and infant baseline neutralization titers. This idea is reinforced by the fact that the maternal baseline serum continues to be effective against the infant viruses for the duration of infection; whereas the ability of the infant serum to neutralize contemporary viruses is reduced after the early timepoints. Moreover, differences in the susceptibility of viral isolates to be neutralized by antibodies was independent of the replication rates, since the 6, 12 and 48-month viral isolates replicated with nearly identical kinetics.

The observed viral diversity increase at 12-months might coincide with the diminution of maternal antibody effectiveness. However, the increasing titer of antibodies beyond 12 month implied the development of *de novo *infant humoral immune responses against the diversifying population. This response, as might be anticipated, is always in reaction to the viral alterations, not in anticipation of it. This conclusion is supported by the finding that despite an apparent failure of the humoral immunity to control HIV-1 replication through neutralizing antibodies at 48 months, infant 1157 mounted an effective neutralizing response to that virus at subsequent timepoints (67 month) (Figure 6C) and this coincided with a decrease in viral diversity (Figure 2). However, the role of cell-mediated immunity in controlling viral replication cannot be determined for this infant since viable cells were not available.

It has been suggested that a more antigenically diverse virus population would correlate to a broader immune reactivity, a slower rate of disease progression [50,51], and selection of neutralization escape mutants in HIV-1 infected individuals, including long-term non-progressors [47,52-54]. Our study, even though with only one mother infant pair, appears to support this hypothesis but further analysis involving a larger number of patients, including rapid and slow progressors, followed longitudinally will be needed to substantiate this observation. A more complete understanding of the mechanisms of humoral immune escape with a more precise definition of the regions in Env where such mutations cluster is likely to impact vaccine design.

It has recently been observed that viruses with shorter V1-V4 Env length, and fewer glycans are more susceptible to neutralizing antibodies, but mediate more efficient transmission in discordant couples [28]. Assuming this concept, one would expect to see a relative lengthening of Env, and an increase in the number of glycans with time. Our analysis of MIP 1157 longitudinally, which was based on C2-V4 sequences, cannot be used for direct comparison for transmission, we did, however, observe increases in variation at PNGS and in sequence length over time. The variation in Env over the follow-up period frequently resulted in the deletion, addition, or relocation of potential N-glycans, suggesting a role of N-glycans for immune selection in the HIV-1 evolution. The hot spots of N-glycan variation were particularly evident in the C2 and C3 regions. Similar changes in potential glycosylation sites have been hypothesized to modify a "glycan shield" for evading neutralizing antibodies [48].

## Conclusion

We have demonstrated that genetic diversification in the infant sequences increased after 12 months, and this coincided with increases in neutralizing antibody titers. In addition, episodes of viral growth and successive immune reactions in the first 5–6 years were observed in this slow progressor infant with delayed onset of AIDS. Longitudinal studies such as the one described here underscore the dynamic and complex interactions of viral populations and immune responses. Whether this pattern of viral host interaction is typical of slow progressing infected infant needs to be further substantiated.

## Methods

### Patient population and sample collection

The mother-infant pair (MIP) 1157 characterized in this study was recruited to investigate the routes of transmission of HIV-1. Venous blood was obtained from the mother before delivery and from the infant within 24 hours of delivery. Follow-up blood specimens were obtained when the pair returned for visits at 6, 12, 18, 24, 29, 36, 48 and 67-months after delivery. The HIV-1 serological status of the mother was determined by two rapid assays, Capillus (Cambridge Biotech, Ireland) and Determine (Abbott laboratories, USA), on the initial blood samples. The positive serological result was confirmed by immunofluorescence assay (IFA), as previously described [55]. The status of HIV-1 infection in the infant was determined by performing viral isolation from the infant's peripheral blood mononuclear cells (PBMC) and by PCR on DNA isolated on the day of birth.

### Viral isolation

HIV-1 was isolated sequentially over a 67-month post-delivery period by standard co-culture procedures. Donor HIV-1-negative PBMC were purified using Lymphoprep (Life Technology). The purified lymphocytes were then propagated in RPMI 1640 medium containing 10% heat-inactivated fetal bovine serum (FBS) and 5 μg/ml of phytohemagglutinin (Sigma) for 40 h before co-culturing with MIP 1157 PBMC or whole blood at a combined final concentration of 2 × 10^6^cells /ml. Equal numbers of fresh uninfected PHA-stimulated PBMC were added to the culture weekly. Virus production was monitored using a commercial ELISA to measure HIV-1 p24 antigen levels (Coulter immunology, FL). Virus stocks were prepared when p24 antigen concentration exceeded 10 ng /ml (about 7–10 days). Viral isolates were recovered from 6-month maternal and 6, 12, 18, 24, 29, and 48-month infant samples

### Biological phenotype

Phenotype, syncytium-inducing (SI) or non-syncytium-inducing (NSI), was determined by infecting MT-2 cells in a 12-well tissue culture plate (5 × 10^5 ^cells / well) with 5 ng of p24 virus stock per well. Cell cultures were observed daily for syncytia formation, over a course of 10 days. Levels of p24 antigen were determined in supernatants collected on day 2, 4, 7, and 10 post-infection. Virus was scored as SI if syncytia formation and increasing level of p24 antigen were observed within the 10-day period, and as NSI if syncytia failed to form within that time.

### Cell tropism

To define the viral tropism, primary monocyte-derived macrophages (MDM), and MT-2 or C8166 T-cell lines were infected with the virus stocks using standard methods. Primary monocytes were obtained from gradient-purified PBMC by adherence to plastic culture dishes [56]. Adherent cells were cultured for 7 to 10 days in RPMI 1640 medium containing 10% FBS and 10 ng/ml of granulocyte-macrophage, colony-stimulating factor (GIBCO) to promote differentiation of monocytes to macrophages. Differentiated macrophages, or T-cell lines, were infected with 5 to 10 ng of HIV-1 p24 antigen per 5 × 10^5 ^cells and incubated for 4 to 5 h at 37°C. Subsequently, the infected cells were washed twice with phosphate buffered saline (PBS) and resuspended in fresh culture medium. Culture supernatants were removed at 3, 7, and 14 days post-infection and assayed for HIV-1 p24 antigen. A culture well was considered virus-positive if increasing level of p24 antigen was observed.

### Chemokine co-receptor usage

Determination of co-receptor usage was carried out using cell lines obtained through the NIH AIDS Research and Reference Reagent Program, Division of AIDS, NIAID, NIH from Dr. Nathaniel Landau that express specific co-receptors (CEMx174-GFP cells [CXCR4], Ghost-CCR5 cells [CCR5] and HOS-CD4-CCR3 cells [CCR3]). PBMC from an individual homozygous for CCR5 mutation Δ32 were obtained from Dr. James Hoxie (University of Pennsylvania). To test for co-receptor usage, the CCR5-Δ32 PBMC and the three co-receptor-specific cell lines were seeded at a density of 1 × 10^6 ^cells/ml into 24-well culture plates. The cells were infected with 5 ng /ml of HIV-1 p24. The infected CEMx174-GFP and Ghost-CCR-5 cells were observed microscopically on day 2–3 post-infection for green fluorescent protein (GFP) expression. Wells exhibiting a count of GFP-expressing cells greater than or equal to 3-fold the negative control wells were scored as positive. Uninfected control wells produced only one to two GFP expressing cells per well. HIV-1 strains SF2 and NL4-3 were used as positive controls for viruses that use CXCR4, and HIV-1 strain SF128A was used as positive control for CCR5 utilization. Positive control viruses consistently gave 7-fold, or greater, GFP- expressing cells than the background control.

Infection of CCR5-Δ32 PBMC and HOS-CD4-CCR3 cells was monitored by measuring HIV-1 p24 antigen production in culture supernatants. HIV-1 p24 was measured at 3 days post-infection for HOS-CD4-CCR3 cells, and at 3, 7 and 10 days post-infection for the CCR5-Δ32 PBMC. Cultures were considered positive for viral growth if more than 100 pg/ml of p24 was detected.

### Viral isolates replication kinetics

Equivalent infectious units, 100 TCID_50_, of the infant viral isolates obtained at 6, 12 and 48-month follow up were added to triplicate wells in a 12-well plate containing 6 × 10^6 ^PHA-stimulated PBMC from a HIV-1 seronegative blood donor. The laboratory isolate 128A was used as a replication kinetics control. After incubation at 37°C for 6 hours, cells were washed 3 times with PBS and refilled with fresh medium. All infected cultures were sampled and supplemented with a 50% volume of fresh culture medium at day 1, 3, 5, 7, 9, 11, 13 and 15. Viral replication kinetics in PBMC was determined by measuring RT units in culture supernatants at day 0, 1, 3, 5, 7, 9, 11, 13 and 15-postinfection. Viruses were lysed using 10% Triton X-100 (1% final concentration) in RPMI medium supplemented with 10% FBS, and RT units was measured using EnzChek Reverse Transcriptase Assay Kit (Invitrogen, Eugene, Oregon). The assay was performed in triplicate.

### Virus neutralization assay

Plasma neutralization activity was determined through infections of TZM-bl cells (NIH AIDS Research and Reference Reagent Program catalogy no. 8129, TZM-bl) as described in Wei et al (2003) with modifications. TZM-bl cells stably express high levels of CD4, CCR5 and CXCR4. The cells contain HIV-1 LTR promoter cassettes that express luciferase and β-galactosidase in response to stimulation with HIV-1 Tat. TZM-bl cells were plated at a density of 6 × 10^3^/well in 96-well tissue culture plates (Falcon) and cultured overnight in DMEM supplemented with 10% FBS. Test plasma was heat-inactivated at 56°C for 30 min, spun at 3,000 × g for 5 min and diluted 1:20, 1:100 and 1:500 in DMEM plus 6% FBS. Viral aliquots of 100 TCID_50_/ml were prepared in DMEM supplemented with 6% FBS and 80 μg/ml DEAE dextran, to a combined total volume of 100 μl. The virus aliquots (100 μl) were combined with 100 μl of the different test plasma dilutions and the mixture was incubated for 1h at 37°C. Following incubation, the virus-plasma mixture was added to TZM- bl cells and incubated at 37°C for two days. Following two washes with PBS, the level of virus infection was measured by luciferase activity. Cells were lysed using Luciferase Assay Reagent (Promega, Madison, WI) and the luciferase activity was measured using a LUMIstar luminometer (BMG Lab Technologies, Offenburg, Germany). The assay was performed in triplicate. Controls included cells infected by virus inoculated with medium or normal human plasma instead of the test plasma. Effective neutralization by the plasma would reduce the level of luciferase versus controls lacking plasma. The luciferase activity in the control wells without plasma was defined as 100 %, and the neutralization titer of the test plasma was calculated relative to this value.

### Polymerase chain reaction, gene cloning, sequencing and subtype identification

Genomic DNA was purified from patient PBMC using the ISOQUICK kit (ORCA Research, Inc.). Primers used for amplification of the subtype C *env *gene were designed based on a reference alignment of all HIV-1 subtypes obtained from the Los Alamos HIV-1 Sequence Database . Nested PCR, as previously described [27], was used to amplify a 617 bp fragment containing the C2-V4 region of the maternal and infant *env *gene from samples collected at birth through 67-month. The amplified fragments were cloned into the pGEM-T Easy vector (Promega) and sequenced in both directions with dideoxy terminators (ABI BigDye Kit).

### Sequence alignment and analyses

Nucleotide sequences were translated and the amino acid sequences aligned using Clustal W, as implemented in BioEdit (Hall 1999)[57], and further refined manually, preserving the reading frame.

Bayesian (BA), maximum likelihood (ML), and neighbor-joining (NJ) phylogenetic analyses were implemented to: 1) determine subtype affiliation (aligning infant clones to a reference panel from the HIV database at Los Alamos National Laboratory ; 2) assess the transmission pattern between 1157 mother and infant (samples collected at birth from the mother and the infant were evaluated), 3) visualize how viral populations change with time (all samples collected from mother and infant were included). Bayesian searches were run in MrBayes version 3.04 [58] for 5 × 10^6 ^generations. Trees were sampled every 1000 generations. Convergence was reached after 1.5 × 10^6^, and a majority rule consensus was built based on the last 3000 trees. ML searches were implemented in Treefinder [59] using a general time-reversible model of nucleotide substitution for each codon position. NJ analyses were performed in PAUP* ver4.10b [60], estimating distances by maximum likelihood, after selecting the best fitting model of nucleotide substitution in Modeltest ver 3.5 [61]. Support for the nodes in ML and NJ was evaluated by running 1000 pseudoreplicates in bootstrap analyses.

### Genetic diversity, temporal structure and estimates of selective strength

Samples were grouped into contemporaneous sets according to the time of collection. Sites with alignment gaps, corresponding to putative insertions or deletions, were excluded from comparisons. Variation in the pattern of genetic diversity for each time-point was explored using MEGA3, [62] and DNAsp ver 4.06 [63], Datamonkey [64] and PAML ver 3.13. [65], and the number and location of putative glycosylation sites (PNGS) were estimated using N-GlycoSite from the Los Alamos National Laboratory database . Viral diversity and viral divergence from the initial population were calculated for each timepoint. Viral diversity estimates were based on *π*, the average nucleotide differences between sequences per site. Changes in viral diversity were used to estimate relative changes in the effective viral population size, assuming a similar mutation rate at each timepoint. Viral divergence was calculated as the average genetic distance to the viral population in the infant at birth.

The relative strength of positive selection was evaluated in a ML framework using PAML ver 3.13. [65]. To estimate the overall dN/dS for each set of contemporaneous sequences we implemented the one-rate codon model described by Yang and Nielsen (1998) [66]. The number of synonymous and non-synonymous substitutions observed per site in the infant Env domains (C2-V4) was calculated in Datamonkey so as to visualize the distribution of synonymous and non-synonymous substitutions along the sequence and to identify regions that accumulate most variation ('hot spots').

## Authors' contributions

HZ carried out the PCR, cloning, and sequencing. FH, GO, HZ and XH performed the sequencing analysis by computer program. JH carried out viral isolation, viral tropism, co-receptor usage and neutralization assay. CK was involved in patient recruitment and follow-up. RR contributed to experimental design and data analysis. HZ, FH, GO, JW and CW participated in the experimental design, data interpretation and writing of the manuscript.
